# Inhibition of VEGF: a novel mechanism to control angiogenesis by *Withania somnifera*’s key metabolite Withaferin A

**DOI:** 10.1186/2193-9616-1-11

**Published:** 2013-07-29

**Authors:** Sanjib Saha, Md Khirul Islam, Jamil A Shilpi, Shihab Hasan

**Affiliations:** Pharmacy Discipline, Life Science School, Khulna University, Khulna, 9208 Bangladesh; Centre for Natural Products and Drug (CENAR), University of Malaya, 50603 Kuala Lumpur, Malaysia; School of Medicine, The University of Queensland (UQ), Brisbane, Queensland Australia; Bioinformatics Lab, Queensland Institute of Medical Research (QIMR), Brisbane, Queensland Australia

**Keywords:** Angiogenesis, VEGF, Withaferin A, *Withania somnifera*, Bevacizumab, DockingServer, SwissDock, Chimera

## Abstract

**Purpose:**

Angiogenesis, or new blood vessel formation from existing one, plays both beneficial and detrimental roles in living organisms in different aspects. Vascular endothelial growth factor (VEGF), a signal protein, well established as key regulator of vasculogenesis and angiogenesis. VEGF ensures oxygen supply to the tissues when blood supply is not adequate, or tissue environment is in hypoxic condition. Limited expression of VEGF is necessary, but if it is over expressed, then it can lead to serious disease like cancer. Cancers that have ability to express VEGF are more efficient to grow and metastasize because solid cancers cannot grow larger than a limited size without adequate blood and oxygen supply. Anti-VEGF drugs are already available in the market to control angiogenesis, but they are often associated with severe side-effects like fetal bleeding and proteinuria in the large number of patients. To avoid such side-effects, new insight is required to find potential compounds as anti-VEGF from natural sources. In the present investigation, molecular docking studies were carried out to find the potentiality of Withaferin A, a key metabolite of *Withania somnifera*, as an inhibitor of VEGF.

**Methods:**

Molecular Docking studies were performed in DockingServer and SwissDock. Bevacizumab, a commercial anti-VEGF drug, was used as reference to compare the activity of Withaferin A. X-ray crystallographic structure of VEGF, was retrieved from Protein Data Bank (PDB), and used as drug target protein. Structure of Withaferin A and Bevacizumab was obtained from PubChem and ZINC databases. Molecular visualization was performed using UCSF Chimera.

**Results:**

Withaferin A showed favorable binding with VEGF with low binding energy in comparison to Bevacizumab. Molecular Docking studies also revealed potential protein-ligand interactions for both Withaferin A and Bevacizumab.

**Conclusions:**

Conclusively our results strongly suggest that Withaferin A is a potent anti-VEGF agent as ascertained by its potential interaction with VEGF. This scientific hypothesis might provide a better insight to control angiogenesis as well as to control solid cancer growth and metastasis.

**Electronic supplementary material:**

The online version of this article (doi:10.1186/2193-9616-1-11) contains supplementary material, which is available to authorized users.

## Background

Angiogenesis is a complex process, where angiogenic endothelial cells undergo a complex process that includes the secretion of metallo-proteases, cell migration, endothelial cell division, and proliferation, including the new blood vessel formation from the endothelium of a pre-existing vasculature (Bruick and McKnight 2001; Cébe-Suarez et al. 2006). Angiogenesis is involved in pathogenesis of various disorders like age-related macular degeneration, proliferative retinopathies, psoriasis, rheumatoid arthritis, and also most the common fatal disorder, solid cancer (Ruggeri et al. 2003; Folkman 1995; Ferrara 2001; Garner 1994). Angiogenesis can be controlled through different anti-angiogenic and pro-angiogenic factors (Drevs et al. 2004; Petrova et al. 1999). Controlling angiogenesis, we can ensure limited growth of solid cancer, because cancer cell will starve without extra supply of nutrients and oxygen (Folkman 1995; Ferrara 2002,2004).

Vascular endothelial growth factor (VEGF) is considered as one of the most vital pro-angiogenic factors involved in tumor angiogenesis (Ferrara 2001; Drevs et al. 2004; Ferrara et al. 2003). VEGF family comprising of glycoproteins designated as VEGF-A, VEGF-B,VEGF-C, VEGF-D, VEGF-E, placental growth factor (PGF), and VEGF-F are involved in the regulation of angiogenesis (Ball et al. 2007; Lee et al. 2010; Otrock et al. 2007; Fayette and Soria 2005).

The endothelial cells are considered to be a novel target for the therapies against cancer cells because of their genomic instability (Frumovitz and Sood 2007; Sood et al. 2011). VEGF is secreted from stabilized over expressed tumor cells, and binds to the receptors on the endothelial cells of existing blood vessels, ultimately leads to new blood vessels formation from existing one, which ensures extra nutrient and blood supply for tumor cell survival, proliferation, and metastasis (Terman and Stoletov 2001). To control angiogenesis, anti-VEGF agents and other VEGF inhibitors are being prescribed in combination with chemotherapy all over the world (Ferrara et al. 2005; Bender and Yamashiro 2011; Morabito and Maio 2006; Carter 2000). The anti-VEGF monoclonal antibody, Bevacizumab, is usually prescribed for the treatment of malignant cell (Ferrara et al. 2005; Bossung and Harbeck 2010). Bevacizumab is used not only in angiogenesis but also in the treatment of breast, colorectal, and prostate cancer (Ferrara et al. 2005; Boige and Malka 2005; Kluetz et al. 2010). But Bevacizumab therapy is associated with serious life threatening side-effects like proteinuria and fetal bleeding, at least in 38% patients (Frumovitz and Sood 2007).

Thus, natural bioactive compounds can be a better way to find new potential anti-VEGF agents with less side-effect to control angiogenesis. In this perspective, in the present in silico pharmacological investigation, *Withania somnifera*’s key metabolite Withaferin A, was studied for their inhibitory activity on VEGF. Different parameters like FullFitness, Gibbs free energy (ΔG), free energy of binding, inhibition constant (Ki), total energy of Van der Waals (vdW) force + hydrogen bond (Hbond) +desolv energy (E_VHD_), electrostatic energy, total intermolecular energy, frequency of binding, interact surface area. Ligand bond, non-ligand bond, hydrogen bond, and its length were studied. A complete interaction profile (hydrogen bonds, polar, hydrophobic, pi-pi, cation-pi and others), and hydrogen bonding interactions (HB plot) were also studied.

## Methods

### Ligand and receptor

**Figure 1:**
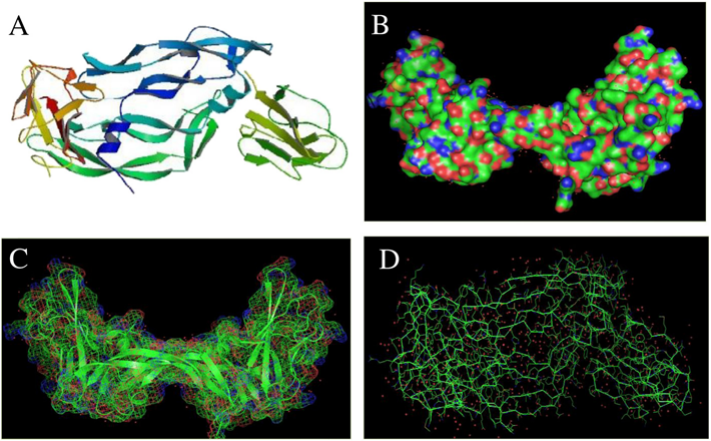
**Structural presentation of VEGF (PDB: 1FLT). (A)** Biological assembly of VEGF, **(B)** Surface structure of VEGF, **(C)** Mesh structure of VEGF, and **(D)** Ribbon structure of VEGF.

**Figure 2:**
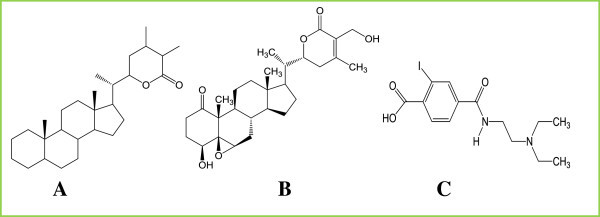
**Structural presentation of ligand molecules. (A)** Withaferin A falls under the family of compounds known as Withanolides, which are naturally occurring C28- steroidal lactones. The basic structure of withanolide skeleton designated as a 22-hydroxyergostan-26-oicacid-26,22-lactone. **(B)** 2D structure of Withaferin A. **(C)** 2D structure of Bevacizumab.

The crystal structure (1.7 Å resolution) of the VEGF in complex with domain 2 of the Flt-1 receptor [PDB: 1FLT], was obtained from the Protein Data Bank (PDB) (Berman et al. 2000). Before Molecular Docking, the protein crystal structure was cleaned by removing the water molecules and hetero atoms. Missing residues (V: 1–12, W: 1–11, X: 1–131, and Y: 1–131) were supplemented to repair the crystal structure. Figure 1 shows the structure of VEGF. The ligand molecules Withaferin A [PubChem: 26759748, ZINC: 08234189] and Bevacizumab [PubChem: 24801581] were retrieved from NCBI-PubChem Compound and ZINC databases (Bolton et al. 2008; Irwin et al. 2012). Figure 2 shows the basic skeleton of Withanolides along with the structure of Withaferin A, and also the structure of Bevacizumab. The Merck molecular force field 94 (MMFF94) was utilized for energy minimization of ligands, and the charge calculation method was Gasteiger. MMFF94 was selected because it is applicable to proteins and other systems of biological significance as well as achieves MM3-like accuracy for small molecules (Halgren 1996a). Moreover, the point of view of the development of MMFF94 guided its intended use in pharmaceutical applications (Halgren 1996a). MMFF94 was developed through *ab initio* techniques of quantum-mechanics at its core and verified by experimental data sets (Halgren [Bibr CR28][Bibr CR29][Bibr CR30][Bibr CR31]). Halgren, pioneered a novel way to more accurately model van der Waals interactions in the development of MMFF94 (Halgren [Bibr CR28][Bibr CR29][Bibr CR30]). The parameterization and performance of MMFF94 for intermolecular interactions has already been validated and it showed parallel performance as OPLS (Optimized Potentials for Liquid Simulations) (Halgren 1996b).

### Molecular docking using DockingServer

Molecular Docking calculations were undertaken using DockingServer (http://www.dockingserver.com) (Bikadi and Hazai 2009). DockingServer is a web-based interface to handle all aspects of molecular docking using AutoDock tools. It can be used for molecular docking and as well as for analysis of results. Moreover, protein and ligand structure can be inputted directly from databases. It has integrated some chemistry software to calculate different parameters of docking study in more efficient way. It was selected because it permits robust molecular docking in more user friendly way with high efficiency.

The MMFF94 force field (Halgren 1996a) was used for the energy minimization of ligand molecules (Withaferin A and Bevacizumab) using DockingServer. Gasteiger charge calculation method was utilized and partial charges were added to the ligand atoms. Non-polar hydrogen atoms were merged, and as well as rotatable bonds were defined.

Molecular Docking calculations were carried out on Withaferin A/ Bevacizumab-VEGF protein model. Necessary hydrogen atoms and solvation parameters were added to the structure with the help of AutoDock tools (Morris et al. 1998). Affinity (grid) maps of 40×40×40 Å (x, y, and z) grid points, and 0.375 Å spacing were automatically generated using the AutoGrid program (Morris et al. 1998). Box center was x: 0.38 Å, y: -2.98 Å and z: 20.51 Å.

Parameter set- and distance-dependent dielectric functions of AutoDock were used for calculating van der Waals and the electrostatic forces, respectively in the Molecular Docking studies.

Molecular Docking simulations were carried out utilizing the Lamarckian genetic algorithm (LGA), and the Solis & Wets local search method (Solis and Wets 1981). Initial position, orientation, and torsions of the ligand molecules (Withaferin A and Bevacizumab) were set on randomly basis. Each docking experiment was derived from 10 different consecutive runs that were set to terminate automatically after a maximum of 250000 energy evaluations. The population size of the docking was set to 150. During the search, a translational step of 0.2 Å, and quaternion and torsion steps of 5 were applied in the current docking.

### Molecular docking using SwissDock

Molecular Docking calculations were performed using SwissDock (http://swissdock.vital-it.ch/) web service based on the docking software EADock DSS (Grosdidier et al. 2011a). This web-based service was selected because it has user friendly interface with the facility to input desired protein and ligand structures directly from databases, modify docking parameters, and visualize most favorable clusters online. Moreover, results can be downloaded and viewed in UCSF Chimera package.

A grid (Box size: 40×40×40 Å and box center: 0.38×-2.98×20.51 for x,y, and z, respectively) was designed in which many binding modes were generated for the most favorable bindings. Simultaneously, their CHARMM energies are estimated on the grid (Grosdidier et al. 2011b). Docking type was accurate and rigid. Each docking experiment was derived from 250 different consecutive runs. The binding modes with the most favorable energies were evaluated with Fast analytical continuum treatment of solvation (FACTS), and clustered. Binding modes were scored using their FullFitness and clustered. Clusters were then ranked according to the average FullFitness of their elements (Grosdidier et al. 2007). Results of the SwissDock were visualized by UCSF Chimera package (Pettersen et al. 2004).

## Results and discussion

**Figure 3:**
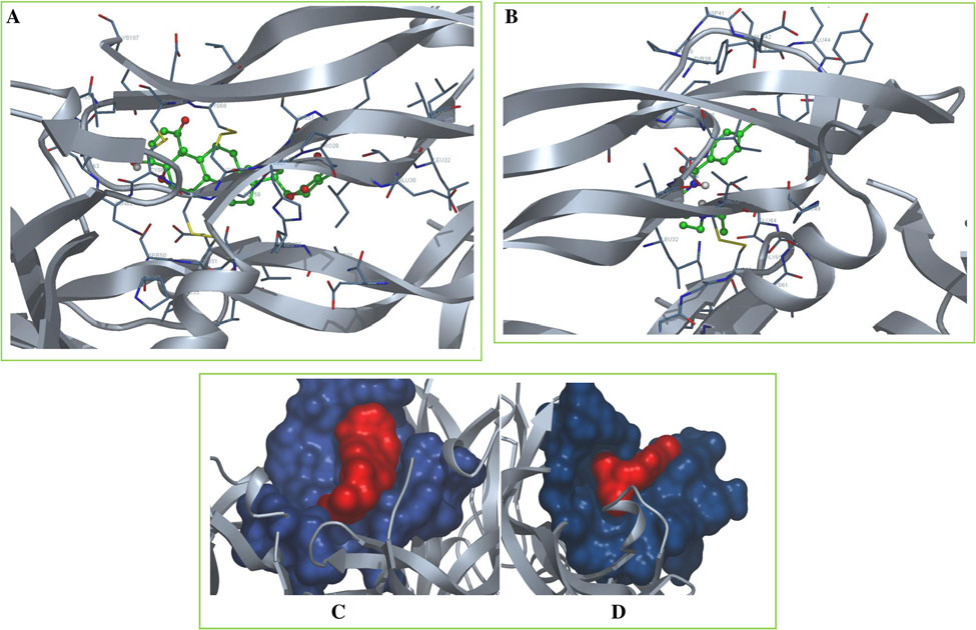
**Visualization of Withaferin A/Bevacizumab-VEGF protein interaction profile by DockingServer.** **(A)** Visualization of Withaferin A-VEGF interaction by DockingServer. Representation of VEGF: cartoon, colour: silver; representation of interacting side chain: cylinder, carbon colour: blue; representation of Withaferin A: ball and stick, carbon colour: green. **(B)** Visualization of Bevacizumab-VEGF interaction by DockingServer. Representation of VEGF: cartoon, colour: silver; representation of interacting side chain: cylinder, carbon colour: blue; representation of Bevacizumab: ball and stick, carbon colour: green. **(C)** Surface visualization of Withaferin A-VEGF interaction by DockingServer.Withaferin A is indicated as red surface and interacting side chain of VEGF is indicated as blue surface. **(D)** Surface visualization of Bevacizumab-VEGF interaction by DockingServer. Bevacizumab is indicated as red surface and interacting side chain of VEGF is indicated as blue surface.

**Figure 4:**
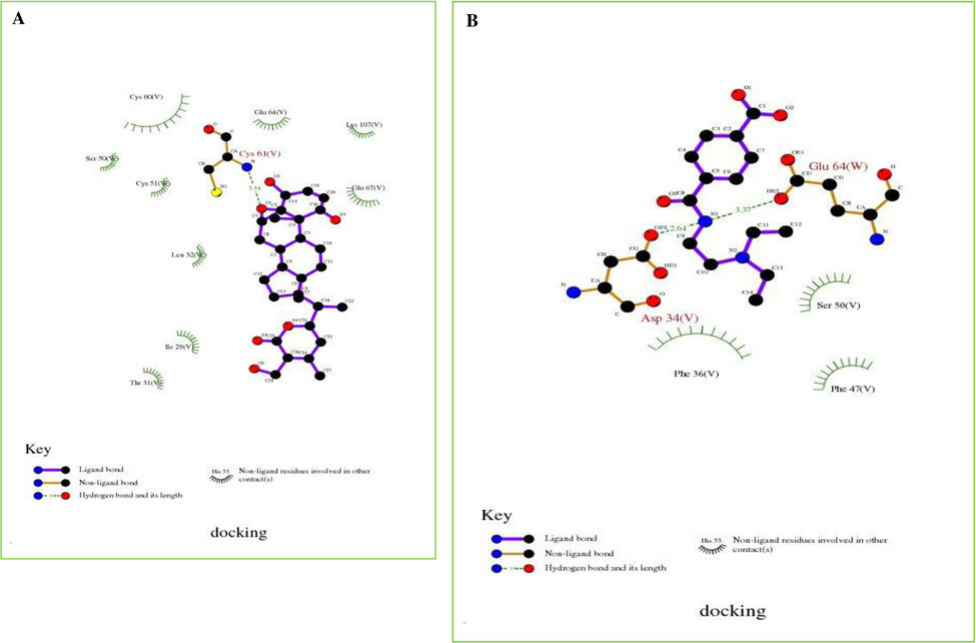
**2D plot of Withaferin A/Bevacizumab-VEGF protein interaction profile by DockingServer.** **(A)** 2D plot of Withaferin A-VEGF interaction by DockingServer. Ligand bond, non-ligand bond, hydrogen bond and its length are mentioned. **(B)** 2D plot of Bevacizumab-VEGF interaction by DockingServer. Ligand bond, non-ligand bond, hydrogen bond and its length are mentioned.

**Figure 5:**
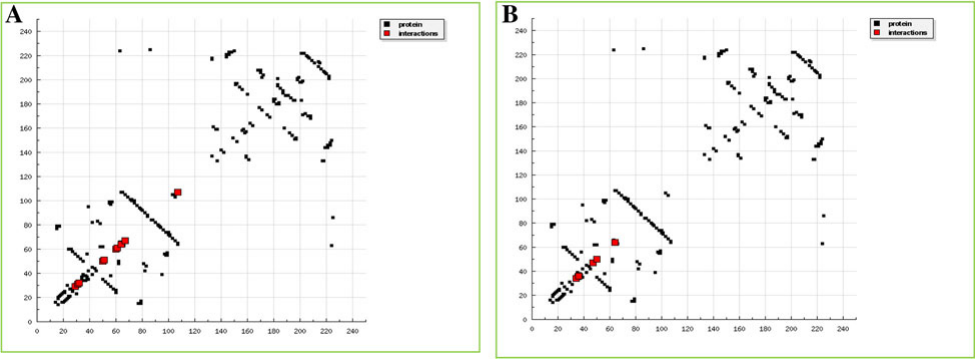
**HB plot of Withaferin A/Bevacizumab-VEGF protein interaction profile by DockingServer.** **(A)** HB plot structure of Withaferin A-VEGF interaction by DockingServer. Interactions with amino acids: 29: Ile, 31: Thr, 32: Leu, 50: Ser, 51: Cys, 60: Cys, 61: Cys, 64: Glu, 67: Glu, and 107: Cys. **(B)** HB Plot structure of Bevacizumab-VEGF interaction by DockingServer. Interactions with amino acids: 34: Asp, 36: Phe, 47: Phe, 50: Ser, and 64: Glu.

In the studies by DockingServer, the parameters of free energy of binding, inhibition constant (Ki), total estimated energy of vdW+Hbond+desolv(E_VHD_), electrostatic energy, total intermolecular energy, frequency of binding, and interact surface area were evaluated to estimate the favorable binding of ligand molecules to the protein. Table 1 shows the complete profile of these parameters of both Withaferin A and Bevacizumab for their interaction with VEGF. For the most favorable binding of Withaferin A, estimated free energy of binding was of -6.09 kcal/mol, and total intermolecular energy was of -7.66 kcal/mol. In case of binding of Bevacizumab, estimated free energy of binding was of -5.59 kcal/mol, and total intermolecular energy was of -7.62 kcal/mol. In comparison to Bevacizumab, Withaferin A exhibited comparatively low free energy of interaction and intermolecular energy. Withaferin A showed the inhibition constant (ki) of 34.53 uM, whereas Bevacizumab showed Ki of 79.65 uM. Figure 3 shows the binding of the legands to the protein. A 2D plot was generated where ligand bond, non-ligand bond, and hydrogen bonds along with their length were mentioned (Figure 4). Decomposed interaction energies of hydrogen bonds, polar, hydrophobic, and other bonds are mentioned in Table 2. Additional file 1 shows the interaction profile of hydrogen bonds, polar, hydrophobic and others. A HB plot (Bikadi et al. 2007 McDonald and Thornton 1994) was generated to mention interactions with different amino acids of the protein (Figure 5).

**Table 1:** **Ligand-protein interaction parameters by DockingServer**

Ligand	Free energy of binding (kcal/mol)	Inhibition constant, Ki (uM)	vdW + Hbond + desolv energy (E_VHD_) (kcal/mol)	Electrostatic energy (kcal/mol)	Total intermolec. energy (kcal/mol)	Frequency	Interact. surface
**Withaferin A**	-6.09	34.53	-7.59	-0.07	-7.66	10%	765.844
**Bevacizumab**	-5.59	79.65	-5.97	-1.65	-7.62	20%	654.544

**Table 2:** **Decomposed interaction energies in kcal/mol by DockingServer**

Ligand	Hydrogen bonds	Polar bonds	Hydrophobic bonds	Other bonds
**Withaferin A**	Cys 61 (0)	Thr 31 (-0.4238), Ser 50 (-0.1325), Glu 64 (0)	Cys 51 (-0.1675), Cys 60 (0)	Leu 32 (-0.1934), Glu 67 (0), Ile 29 (0), Lys 107 (0)
**Bevacizumab**	Glu 64 (-1.0692); Asp 34 (0)	-----	Phe 36 (0)	Phe 47 (0); Ser 50 (0)

**Figure 6:**
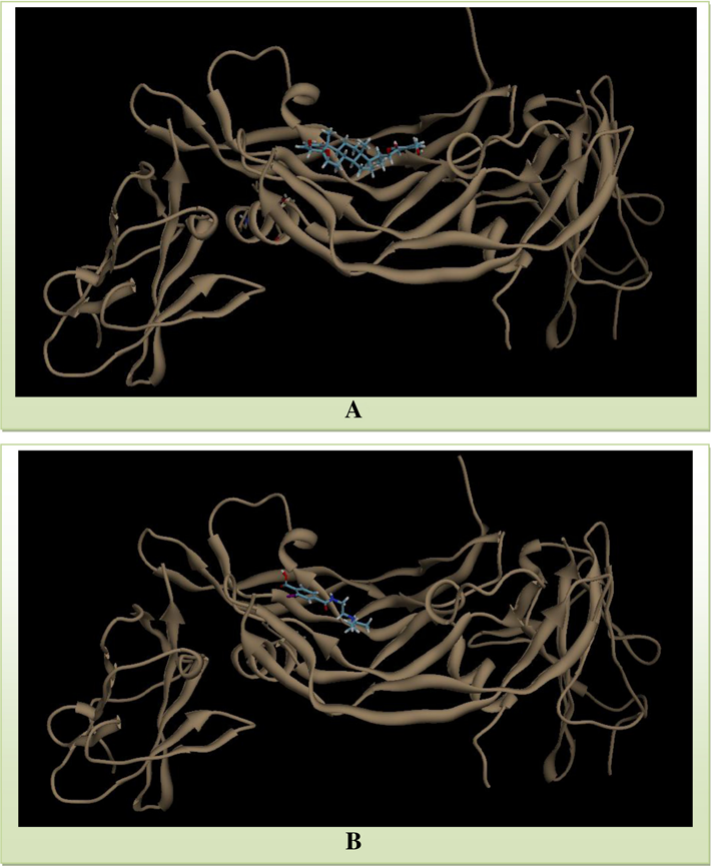
**Visualization of Withaferin A/Bevacizumab-VEGF protein interaction profile by SwissDock.** **(A)** Visualization of Withaferin A-VEGF interaction by SwissDock. **(B)** Visualization of Bevacizumab-VEGF interaction by SwissDock. Visualization is performed using UCSF Chimera.

In the studies by SwissDock, FullFitness and Gibbs free energy (ΔG) of each run (250 runs) of the docking were evaluated. Favorable binding modes were scored based on FullFitness and cluster formation. Ranking of the cluster was performed using the value of FullFitness. Tables 3 and 4 shows the clustering results obtained from the docking of the ligands into VEGF protein. Withaferin A showed FullFitness of -1948.69 kcal/mol and estimated ΔG of -7.24 kcal/mol for the most favorable interaction, whereas Bevacizumab showed FullFitness of -2221.84 kcal/mol and ΔG of -7.56 kcal/mol. Figure 6 shows the visualization of the most energetically favorable binding of the legands into the protein VEGF.

**Table 3:** **Clustering results obtained from the docking of Withaferin A into VEGF by SwissDock**

Receptor	No. of SwissDock clusters	Cluster rank	FullFitness (kcal/mol)	Estimated ΔG (kcal/mol)
VEGF	30(250 runs)	1	-1948.69	-7.24
		2	-1947.96	-7.86
		3	-1946.36	-6.83
		4	-1945.21	-7.51
		5	-1944.75	-6.79

**Table 4:** **Clustering results obtained from the docking of Bevacizumab into VEGF by SwissDock**

Receptor	No. of SwissDock clusters	Cluster rank	FullFitness (kcal/mol)	Estimated ΔG (kcal/mol)
VEGF	30(250 runs)	1	-2221.94	-7.56
		2	-2221.50	-7.78
		3	-2221.25	-7.12
		4	-2220.26	-7.46
		5	-2219.98	-7.20

Based on the results of docking studies, it has been clearly expressed that Withaferin A showed favorable binding with VEGF, and the results were highly comparable with the commercially available drug Bevacizumab. VEGF, as an angiogenic protein stimulates the process of angiogenesis through chemical stimulation. Withaferin A shows favorable binding with VEGF, which can be potential way to prevent chemical stimulation of VEGF to induce angiogenesis process in hypoxic condition of the solid tumors. Moreover, VEGF is considered as one of the most vital pro-angiogenic factors involved in tumor angiogenesis (Moreira et al., 2007). VEGF increases vascular permeability which propagates tumor dissemination with the supply of sufficient oxygen and nutrients (Moreira et al., 2007). Inhibition of VEGF can prevent aggressive tumor angiogenesis which prevents the supply of oxygen and nutrients, necessary for propagation of tumor; ultimate outcome is the retardation of tumor growth.

In hypoxic condition, up-regulation of hypoxia inducible factor (HIF1), provokes VEGF growth factor, which in association with other cytokines, induces neovascularization of tumors and creates favorable conditions to grow beyond the size limitation (Martinez et al., 2003). For the first time, VEGF was accused in angiogenesis when it was identified as a growth factor secreted by solid tumor cells, which caused the hyperpermeability of normal blood vessels (Senger et al., 1983). Though VEGF presents in almost every type of tumor, but it is high in concentration in the tumor blood vessels and hypoxic area of the tumor. VEGF binds with specific receptor, so inhibition of VEGF receptor or inhibition of VEGF to bind with the receptor can definitely retard the growth of solid tumors (Millauer et al., 1996). It has already been experimented that the injection of an antibody VEGF, suppresses the growth of solid tumors of human fibrosarcoma cell line HT-1080 (Asano et al., 1995).

Recently, in cancer therapy, new strategies show the clinical relevance of inhibiting VEGF when the angiogenesis process is exaggerated, particularly in pathological angiogenesis (Olsson et al., 2006). However, such therapies in the long term management of cancer can hamper the survival of blood vessels in the healthy tissues (Olsson et al., 2006). So, in the development of the inhibitor of VEGF, it is vital to preserve the pathways associated with the survival of blood vessels necessary to conduct normal physiological function and development (Olsson et al., 2006). In addition, VEGF is essential for transporting oxygen, nutrients, and the removal of carbon dioxide and metabolic end products from cells, tissues, and organs to accomplish normal physiological phenomena (Cines et al., 1998). In tumor therapy, while using VEGF inhibitor, we have to calculate risk benefit ratio to validate the therapy.

Throughout the study, Withaferin A was better VEGF inhibitor than Bevacizumab in aspect of binding and affinity. Clinically, Bevacizumab is the most successful VEGF-neutralizing agent which was approved by the United States Food and Drug Administration (FDA) in the year of 2004 (Olsson et al., 2006). In combination with chemotherapy, Bevacizumab prolongs the survival rate of patients with solid tumor (Olsson et al., 2006). It has been often regarded that anti-VEGF drugs normalize the tumor blood vessels, which ensures more efficient delivery of the chemotherapy drugs in the tissue (Jain 2005). In addition, another anti-VEGF drug, Ranibizumab, derived from the same mouse antibody as Bevacizumab, playing their role in controlling angiogenesis through the inhibition of a number of subtypes of VEGF (Haberfeld 2009). Withaferin A showed more promising activity than Bevacizumab in molecular docking studies which leads the potential of Withaferin A, as a promising VEGF inhibitor with lower side-effects because of its natural origin. Though only VEGF inhibition can’t inhibit tumor angiogenesis because there are many other endogenous anti-angiogenic factors available in our physiological system, but it can definitely retard aggressiveness of the tumor angiogenesis in some extent (Roskoski 2007).

## Conclusions

The protein-ligand interaction studies play a vital role in the structure based drug design in dry lab. VEGF is one of the most attractive topics in cancer biology, biochemistry, and pharmacology, and in the recent years the number of studies focusing on its inhibition has increased manifolds. Present study, has given a new insight to inhibit VEGF with the key metabolite, Withaferin A of *Withania somnifera*. Further investigations like QSAR studies are required to study semi-synthetic derivatives of Withaferin A to get more favorable interaction into VEGF.

## Electronic supplementary material

Additional file 1: Table S1: (A) Withaferin A-VEGF Interaction profile by DockingServer. (B) Bevacizumab-VEGF Interaction profile by DockingServer. (DOC 318 KB)

## Abbreviations

CHARMM: Chemistry at HARvard macromolecular mechanics
EVHD: Desolv energy
FACTS: Fast analytical continuum treatment of salvation
ΔG: Gibbs free energy
HB: Hydrogen bonding
Hbond: Hydrogen bond
HIF1: Hypoxia inducible factor
Ki: Inhibition constant
LGA: Lamarckian genetic algorithm
MMFF94: Merck molecular force field 94
OPLS: Optimized potentials for liquid simulations
PDB: Protein data bank
PGF: Placental growth factor
UCSF: University of California, San Francisco
VEGF: Vascular endothelial growth factor
vdW: van der Waals.
