# Supplementary material for: Inhibition of VEGF: a novel mechanism to control angiogenesis by Withania somnifera’s key metabolite Withaferin A
Source: In Silico Pharmacol. 2013 Jul 29;1:11. doi: 10.1186/2193-9616-1-11 (PMC4230651; doi:10.1186/2193-9616-1-11)

**Table S1 (A)Withaferin A-VEGF Interaction profile by DockingServer**


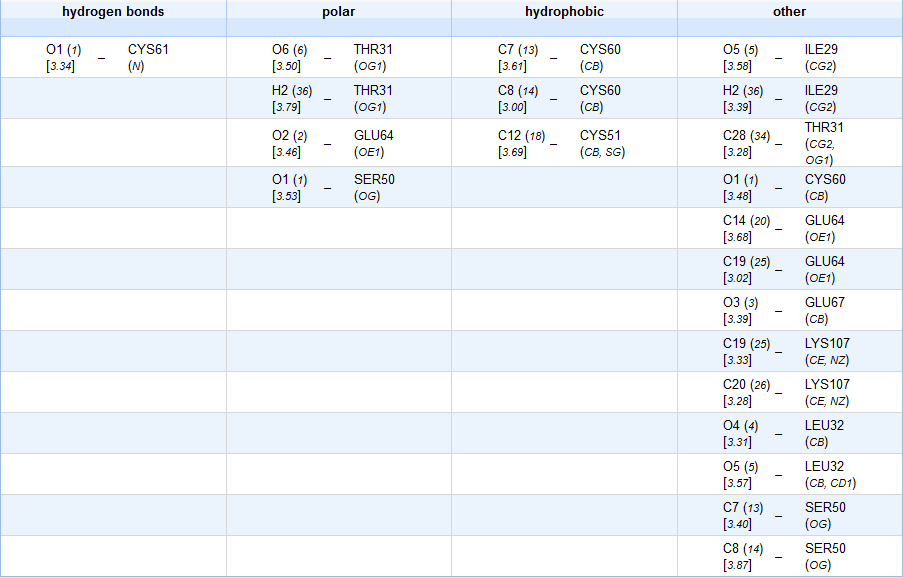


**Table S1 (B) Bevacizumab-VEGF Interaction profile by DockingServer**


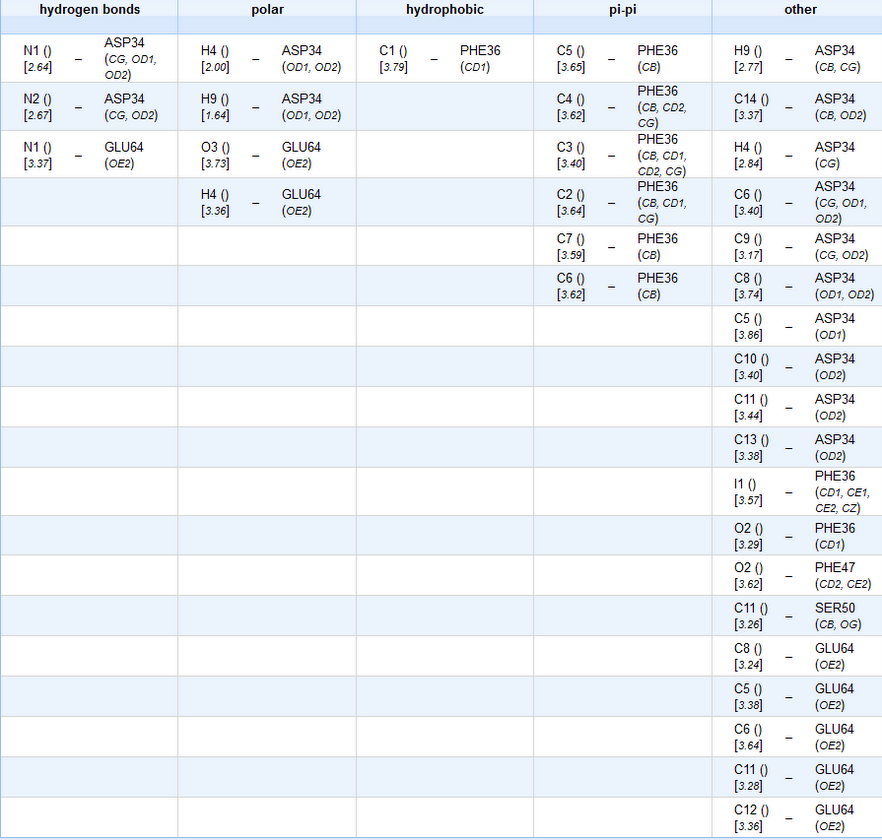

Supplement: Supplementary file 1 — Additional file 1: Table S1: (A) Withaferin A-VEGF Interaction profile by DockingServer. (B) Bevacizumab-VEGF Interaction profile by DockingServer. (DOC 318 KB) [file 40203_2013_11_MOESM1_ESM.doc]
